# Associations of age at menopause, bilateral oophorectomy, hysterectomy and hormone replacement therapy with glycaemia and risk of dementia: a study based on the population-based UK Biobank cohort

**DOI:** 10.1136/bmjph-2024-002120

**Published:** 2025-07-27

**Authors:** Anouk Geraets, Katherine Ford, Patrick May, Emma Kidd, Anja Leist

**Affiliations:** 1 Department of Social Sciences, University of Luxembourg, Esch-sur-Alzette, Luxembourg; 2 Department of Psychology, Carleton University, Ottawa, Ontario, Canada; 3 Luxembourg Centre for Systems Biomedicine, University of Luxembourg, Esch-sur-Alzette, Luxembourg; 4 School of Pharmacy and Pharmaceutical Sciences, Cardiff University, Cardiff, UK

**Keywords:** Epidemiologic Methods, Female, Mental Health, Preventive Medicine

## Abstract

**Introduction:**

Menopausal factors, including age at menopause, bilateral oophorectomy, hysterectomy and hormone replacement therapy (HRT), have been associated with dementia risk, which might be due to increased glucose levels after menopause. This study investigated the associations of these menopausal factors with glycaemia and dementia risk, and whether glycaemia mediates the association of menopausal factors with dementia risk.

**Methods:**

We used longitudinal data from the population-based UK Biobank cohort (n=147 119 women; mean±SD age 55.2±8.0 years at baseline). Menopausal status, bilateral oophorectomy, hysterectomy, HRT and age at natural menopause, surgery and HRT were self-reported. Glycaemia was measured with fasting plasma glucose and haemoglobin A1c (HbA1c) levels. Dementia diagnoses were ascertained from hospital records. Cox proportional hazards regression analyses tested the associations between menopausal factors, glycaemia and dementia risk. Causal mediation models were used to test mediation.

**Results:**

After a mean follow-up of 12.5±1.6 years, 1385 participants had incident dementia. Though there was a direct effect of bilateral oophorectomy, hysterectomy, lifetime HRT and age at natural menopause and surgery on fasting plasma glucose and HbA1c levels, only age at natural menopause (HR=0.97 (95% CI 0.96 to 0.99) per year and HR=1.31 (95% CI 1.08 to 1.60) for early natural menopause) and lifetime HRT in women with natural menopause (HR=1.13 (95% CI 1.00 to 1.27)) were associated with dementia risk. Causal mediation analyses showed that up to 4.7% of the total effect of age at natural menopause on dementia risk was mediated by HbA1c levels, while both fasting plasma glucose and HbA1c affected the increased dementia risk for lifetime HRT in women with natural menopause.

**Conclusions:**

We observed associations between bilateral oophorectomy, hysterectomy, lifetime HRT and age at natural menopause and surgery with glycaemia. An earlier age at natural menopause was associated with increased dementia risk, and HbA1c marginally mediated this association. Inconsistent associations between HRT and dementia risk require further research.

**WHAT IS ALREADY KNOWN ON THIS TOPIC**

- Menopausal factors may contribute to the increased risk for dementia in women compared with men through hyperglycaemia.

**WHAT THIS STUDY ADDS**

- Up to 4.7% of the total effect of age at natural menopause on dementia risk is mediated by haemoglobin A1c levels, while no mediation by fasting plasma glucose is found.
- Associations of lifetime hormone replacement therapy with glycaemia and risk for dementia depend on menopausal type.

**HOW THIS STUDY MIGHT AFFECT RESEARCH, PRACTICE OR POLICY**

- Monitoring the metabolic profile of women who have an increased risk for dementia may not only prevent hyperglycaemia but may also decrease dementia risk.
- The association of hormone replacement therapy and dementia risk is complex and requires further research to consider age, menopausal type and vasomotor symptoms.

## Introduction

 Globally, the prevalence of dementia among those aged 65 years has been reported to be 1.46 times higher for women compared with men.^1^ The rapid decline in oestrogenss and progesterone after menopause in women, compared with the more gradual age-related decline in testosterone in men, may contribute to increased dementia risk in women compared with men.^2 3^ This may especially be the case after surgical menopause in which both ovaries (bilateral oophorectomy) and/or the uterus (hysterectomy) are removed.^4^ Early age at menopause (before 45 years of age according to the UK National Health Service (NHS)^5^ and surgical menopause have been related to increased dementia risk.^6^,^8^ However, another systematic review and meta-analysis did not find associations between age at natural or surgical menopause and dementia risk but did find indications for better cognitive performance and delayed cognitive decline with advanced age at menopause, supporting a link between female hormone deficiency and cognitive ageing.^9^

The timing hypothesis suggests that the effects of hormone replacement therapy (HRT) on the brain depend on age and time window at the initiation of treatment, being beneficial when initiated early after menopause but may become neutral or detrimental if HRT is initiated later in life when vascular or degenerative lesions are present in the brain.^10 11^ However, recent observational studies challenge the timing hypothesis by finding harmful effects.^12^,^16^ Cardiometabolic risk factors have been proposed as an underlying mechanism for the association between HRT and increased dementia risk,^11^ with HRT shown to be beneficial for glycaemia levels.^17 18^

Metabolic changes during the transition to menopause may contribute to dementia risk via hyperglycaemia.^19^ The brain has a high and continuous glucose demand that, in contrast to other tissues, cannot replace its metabolism with other substrates in case of deficiency of glucose.^20^ Consequently, disruptions in glucose metabolism can directly impact brain activity and neuronal plasticity. Both early age at menopause and surgical menopause have been associated with increased risk of type 2 diabetes mellitus (T2DM),^21 22^ where insulin insufficiency interrupts glucose metabolism leading to higher blood glucose levels. Results of a dose-response meta-analysis have shown that the risk of T2DM was reduced by 10% with each 5-year increment in age at natural or surgical menopause.^21^ Furthermore, findings from the China Kadoorie Biobank study have shown that women with natural perimenopausal or postmenopausal status have an increased risk to develop T2DM compared with premenopausal women, with both earlier and later age at natural menopause associated with T2DM risk.^23^ In a study of women aged 30–49 years, hysterectomy was associated with a 1.4 times higher risk of T2DM, while hysterectomy with oophorectomy was not related to an increased risk of T2DM.^22^ However, the study participants’ age range was younger than the worldwide average age of natural menopause of 45–55 years.^24^ Important to note, all studies used categorical instead of continuous values of glycaemia. In addition to T2DM being associated with an increased dementia risk,^25 26^ higher average levels of glycaemia regardless of a diabetes diagnosis have also been related to an increased dementia risk.^27^ This further supports hyperglycaemia as an underlying mechanism involved in the aetiology of dementia.^19^ Given that hyperglycaemia is a worldwide increasing problem,^28^ it is important to investigate its contribution to the association of menopausal factors with dementia risk.

The aim of this study was to investigate the associations of bilateral oophorectomy, hysterectomy, age at natural menopause and surgery, and lifetime and initiation of HRT with fasting plasma glucose and haemoglobin A1c (HbA1c) levels and dementia risk. In addition, we assessed whether glycaemia mediated the associations of these menopausal factors with dementia risk. We hypothesised that (1) bilateral oophorectomy, hysterectomy and early age at natural menopause and surgery are associated with higher levels of glycaemia and increased dementia risk; (2) lifetime HRT and initiation of HRT close to menopause are associated with lower levels of glycaemia but associations with dementia risk are inconsistent depending on age and type of menopause or surgery and (3) higher levels of glycaemia partly mediate the association between these menopausal factors and dementia risk.

## Materials and methods

### Study population and design

**Figure 1:**
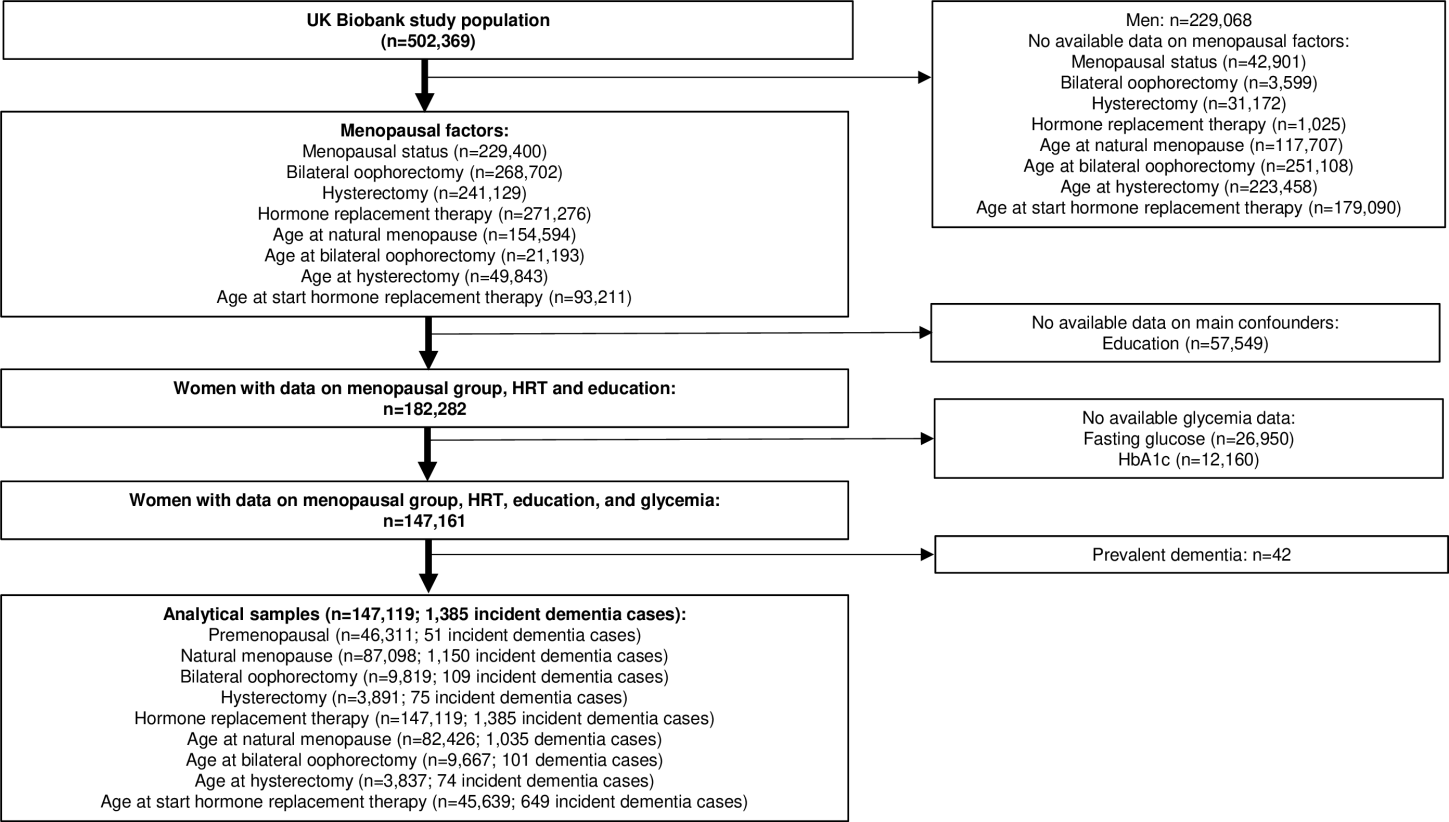
Flow chart of the study population. HbA1c, haemoglobin A1c; HRT, hormone replacement therapy.

We used longitudinal data from the UK Biobank, a population-based cohort study in the UK that collected baseline data from 502 536 participants aged 40–69 years between 2006 and 2010.^29^ This study included follow-up data for dementia risk until 6 November 2021. Figure 1 shows the flow chart for obtaining the study population. Women who had missing data on menopausal status, bilateral oophorectomy, hysterectomy, HRT, age of surgery and menopause in case of surgical intervention, and those who were not sure or preferred not to answer, were excluded from the analyses. Of the 502 536 participants, 239 831 women could be categorised into one of the four menopausal groups (premenopausal, natural menopause, bilateral oophorectomy or hysterectomy). Age at natural menopause, age at bilateral oophorectomy, age at hysterectomy and age at start of HRT were reported by 154 594, 21 193, 49 843 and 93 211 women, respectively. Data on education were unavailable among 57 549 participants. Of the 182 282 women with data on menopausal group and the main confounders, 147 161 women had complete data on glycaemia. The analytical sample (n=147 119; 1385 incident dementia cases) excluded participants with prevalent dementia (n=42) and included 46 311 premenopausal women (n=51 incident dementia cases), 87 098 women who underwent natural menopause (n=1150 incident dementia cases), 9819 women who underwent bilateral oophorectomy prior to menopause (n=109 incident dementia cases) and 3891 women who underwent hysterectomy prior to menopause (n=75 incident dementia cases). Age at natural menopause was available in 82 426 participants (n=1035 dementia cases), age at bilateral oophorectomy was available in 9667 participants (n=101 dementia cases), age at hysterectomy in 3837 participants (n=74 incident dementia cases) and age at start HRT in 3837 participants (n=74 incident dementia cases).

### Menopausal factors

Baseline data on menopausal status, bilateral oophorectomy and hysterectomy, including age, were self-reported (see online supplemental material for the exact wording). Female participants were divided into four groups: (1) premenopausal women without surgical interference, (2) women who underwent natural menopause without surgical interference prior to menopause but probably surgical interference after natural menopause, (3) women who underwent a bilateral oophorectomy (with or without a hysterectomy) prior to menopause and (4) women who underwent a hysterectomy (without bilateral oophorectomy) prior to menopause. The average age at natural menopause in our study sample was 50.3 years (table 1). Therefore, we compared women who underwent bilateral oophorectomy/hysterectomy and were aged below 50 years at baseline to premenopausal women, and women who underwent bilateral oophorectomy/hysterectomy aged 50 years or older at baseline to women who underwent natural menopause. Age at natural menopause, bilateral oophorectomy and hysterectomy were treated as both a continuous variable, as well as a categorical variable for early menopause (< 45 years of age). HRT was assessed with the question: “Have you ever used hormone replacement therapy (HRT)?” and categorised into lifetime history of HRT (yes/no). To test the timing hypothesis,^10 11^ age at HRT initiation was categorised into close to menopause (initiation <61 years or initiation <11 years from menopause for women with menopause at age >44 years and initiation <52 years for women with menopause at age <45 years) and later life (initiation >60 years or initiation >10 years from menopause for women with menopause at age >44 years and initiation >51 years for women with menopause at age <45 years).^30^

**Table 1:** Baseline characteristics of study population

Characteristic	Incident dementia		P value	Available data (n=)
	No (n=145 734)	Yes (n=1385)		
Demographics				
Age (years)	55.1±8.0	63.7±5.3	<0.001	147 119
High educational attainment, n (%)	60 588 (41.6)	467 (33.7)	<0.001	147 119
Townsend deprivation index*	2.08±0.33	2.10±0.34	0.041	146 943
Menopausal factors				
Premenopausal, n (%)	46 260 (31.7)	51 (3.7)	<0.001	147 119
Natural menopause, n (%)	85 948 (59.0)	1150 (83.0)	<0.001	147 119
Bilateral oophorectomy, n (%)	9710 (6.7)	109 (7.9)	0.073	147 119
Hysterectomy, n (%)	3816 (2.6)	75 (5.4)	<0.001	147 119
Age of natural menopause (years)	50.3±4.5	50.0±5.0	0.102	82 426
Early natural menopause (< 45 years), n (%)	7588 (9.3)	111 (10.7)	0.124	82 426
Age at bilateral oophorectomy (years)	45.8±6.7	46.2±7.2	0.486	9667
Early bilateral oophorectomy (< 45 years), n (%)	3500 (36.6)	35 (34.7)	0.688	9667
Age hysterectomy (years)	41.4±6.4	41.5±7.6	0.961	3837
Early hysterectomy (< 45 years), n (%)	2578 (68.5)	55 (74.3)	0.286	3837
Ever used HRT, n (%)	49 065 (33.7)	779 (56.3)	<0.001	147 119
Age start HRT (years)	47.7±5.3	48.0±5.6	0.063	45 639
Initiation HRT close to menopause *, n (%)	34 841 (98.7)	531 (98.0)	0.127	35 836
Glycaemia				
Fasting plasma glucose (mmol/L)	5.0±1.0	5.3±1.5	<0.001	147 119
HbA1c (mmol/mol)	35.4±5.6	38.0±8.5	<0.001	147 119
Cardiometabolic risk factors				
Waist circumference (cm)	83.6±12.3	85.9±13.3	<0.001	146 854
Triglyceride-to-HDL ratio	1.03±0.77	1.13±0.81	<0.001	146 996
Lipid-modifying medication, n (%)	14 278 (9.8)	372 (26.9)	<0.001	147 119
Systolic blood pressure (mm Hg)	135.5±19.9	144.2±20.6	<0.001	137 682
Diastolic blood pressure (mm Hg)	80.4±10.6	81.0±10.6	0.046	137 683
Antihypertensive medication, n (%)	13 062 (9.0)	181 (13.1)	<0.001	147 119
Behavioural risk factors				
Alcohol use frequency days per week (0/1–4/5–7), n (%)	49 292/70 434/25 931 (33.8/48.4/17.8)	595/536/253 (43.0/38.7/18.3)	<0.001	147 041
Current smoker, n (%)	11 514 (7.9)	114 (8.2)	0.654	147 058
Moderate physical activity (min/day) †	3.7±0.9	3.8±0.9	<0.001	111 261
Ever had mental health problems, n (%)	58 082 (40.1)	619 (45.0)	<0.001	146 182
Genetic risk				
Standardised polygenic risk scores for dementia	0.04 (0.99)	0.77 (1.20)	<0.001	145 810

Data are presented as means±SD or number (%).

* Initiation <61 years or initiation <11 years from menopause for women with menopause at age >44 years or initiation <52 years for women with menopause at age <45 years.

† Values are log transformed because of skewed distribution.

HbA1c, haemoglobin A1c; HDL, high-density lipoprotein; HRT, hormone replacement therapy.

### Glycaemia

Baseline glycaemia was measured by levels of fasting plasma glucose and HbA1c (table 1). Fasting plasma glucose in mmol/L was measured by hexokinase analysis on a Beckman Coulter AU5800 at baseline (analytical range 0.6–45 mmol/L). HbA1c (mmol/mol), providing information about a 3-month average blood glucose level, was measured using HPLC from blood samples on a Bio-Rad VARIANT II Turbo (analytical range of 15–184 mmol/mol). Glycaemic measurements were standardised into z-scores.

### Dementia risk

Incident all-cause dementia was ascertained from ICD-9 and ICD-10 (International Classification of Diseases, 9th and 10th revisions) codes of hospital inpatient data that contained data on admissions and diagnoses (England: Hospital Episode Statistics; Scotland: Scottish Morbidity Record; and Wales: Patient Episode Database) and death records (England and Wales: NHS Digital; Scotland: Information and Statistics Division), as described previously.^31^ Dementia diagnosis has high validity in English hospital records (sensitivity, 78%; specificity, 92%)^32^ and in Scottish routine data (positive predictive value, 83%).^31^ Follow-up data for dementia risk until 6 November 2021, was included with a mean follow-up duration of 12.5±1.6 years.

### Statistical analyses

Statistical analyses were performed in Stata V.18 (StataCorp). Cox proportional hazards regressions were used to assess the associations of menopausal factors and markers of glycaemia with time to dementia, resulting in HRs and their 95% CIs. The failure event was incident dementia. Time-in-study was used as the time scale. Follow-up time was calculated from baseline assessment to incidence of dementia, death, dropout or 6 November 2021, whichever came first. Linear regression analyses were used to assess the associations of menopausal factors with fasting plasma glucose and HbA1c. Causal mediation was tested using the mediate command. Probit models were used to estimate the total effect of menopausal factors on incident dementia, and to decompose total effects into direct and indirect effects via the markers of glycaemia. Age at natural or surgical menopause was standardised and an SD of +1 was compared with an SD of −1. All analyses were adjusted for age at baseline, educational attainment and history of HRT. A binary variable for high educational attainment was created in which university/college degree was compared with A levels/O levels/certificate of secondary education/national vocational qualifications (or equivalent). A two-sided p<0.05 was considered statistically significant. Analyses on HRT were stratified by type of menopause (natural and surgical).^11^

Several additional analyses were performed. As cardiometabolic and behavioural factors might be on the causal pathway from menopausal factors to brain ageing,^33^,^35^ we additionally adjusted for baseline cardiometabolic (waist circumference, triglyceride-to-high-density lipoprotein (HDL) ratio, systolic blood pressure, and use of cholesterol, or blood pressure-lowering medication) and self-reported behavioural (alcohol use frequency (5–7/1–4/0 times per week), current tobacco use (yes/no), minutes of daily moderate physical activity and ever visited a general practitioner because of mental health problems (yes/no)) factors in additional analyses.^36^ To consider baseline area-level socioeconomic deprivation, analyses were additionally adjusted for the Townsend Deprivation Index.^37^ Because of its skewed distribution and score range from −6.3 to 11, a value of 10 was added to the Townsend Deprivation Index, whereafter it was log-transformed. To examine whether genetic risk for dementia affects the associations of menopausal factors with dementia risk, we additionally adjusted for polygenic risk score for Alzheimer’s disease (AD). Interactions of menopausal factors with polygenic risk score for AD on dementia risk were tested to investigate whether menopausal factors had similar effects on dementia risk in those with a low or high genetic predisposition. Polygenic risk scores for AD were obtained from UK Biobank (data field: 26206, Standard PRS for AD) and calculated as the z-standardised weighted sum of the number of prevalent alleles at each AD-related single nucleotide polymorphism, including APOE genotype as described previously.^38^

## Results

Participants excluded from analyses because of unavailable data were older, less educated, more deprived, more often developed dementia, had a worse cardiometabolic risk profile, were more likely to be postmenopausal, had undergone bilateral oophorectomy or hysterectomy, had used HRT, had undergone natural menopause or surgery at an earlier age and initiated HRT later in life compared with participants included in our analyses (online supplemental eTable 1).

After a mean follow-up period of 12.5±1.6 years, 1385 participants had incident dementia. Participants who developed dementia were more likely to be older, less educated, more deprived, postmenopausal, had undergone hysterectomy prior to menopause, had a history of HRT, higher levels of glycaemia, a worse cardiometabolic risk profile and more lifetime mental health problems (table 1). Women who underwent bilateral oophorectomy or hysterectomy were more likely to be lower educated, have a higher waist circumference, higher triglyceride-to-HDL ratio, use lipid-modifying or antihypertensive medication and have had lifetime mental health problems compared with women who underwent natural menopause. In addition, women who had undergone bilateral oophorectomy more often used HRT (online supplemental eTable 2).

### Association of menopausal factors and glycaemia with dementia risk

The associations of menopausal factors and glycaemia with dementia risk are shown in table 2. As the number of incident dementia cases was too small for participants who underwent bilateral oophorectomy (n=5) and hysterectomy (n=2) prior to menopause and aged below 50 years at baseline, we only compared dementia risk for women who underwent these surgeries prior to menopause aged ≥50 years at baseline to women who underwent natural menopause without surgical interference. Women aged ≥50 years at baseline who underwent bilateral oophorectomy (HR=0.87 (95% CI 0.71 to 1.07)) or hysterectomy (HR=1.10 (95% CI 0.86 to 1.39)) prior to menopause did not differ in their dementia risk compared with women who underwent natural menopause. Older age at natural menopause was associated with a decreased dementia risk (HR=0.97 (95% CI 0.96 to 0.98) per year) and early natural menopause with an increased dementia risk (HR=1.31 (95% CI 1.08 to 1.60)), while age at bilateral oophorectomy (HR=0.97 (95% CI 0.95 to 1.00) per year), early bilateral oophorectomy (HR=1.44 (95% CI 0.95 to 2.17)), age at hysterectomy (HR=0.98 (95% CI 0.95 to 1.02) per year) and early hysterectomy (HR=1.60 (95% CI 0.95 to 2.71)) were not associated with dementia risk.

**Table 2:** Associations of menopausal factors and markers of glycaemia with dementia risk

	Number of participants in analysis (number of dementia cases)	Incident dementia HR (95% CI)	P value
Menopausal factors			
Bilateral oophorectomy aged ≥50 years*	95 711 (1254)	0.87 (0.71 to 1.07)	0.180
Hysterectomy aged ≥50 years*	90 739 (1223)	1.10 (0.86 to 1.39)	0.447
Age of natural menopause (years)	82 426 (1035)	**0.97 (0.96 to 0.98**)	**<0.001**
Early natural menopause	82 426 (1035)	**1.31 (1.08 to 1.60**)	**0.007**
Age at bilateral oophorectomy (years)	9667 (101)	0.97 (0.95 to 1.00)	0.061
Early bilateral oophorectomy	9667 (101)	1.44 (0.95 to 2.17)	0.087
Age hysterectomy (age)	3837 (74)	0.98 (0.95 to 1.02)	0.301
Early hysterectomy	3837 (74)	1.60 (0.95 to 2.71)	0.079
Markers of glycaemia			
Fasting plasma glucose (per 1 SD)	147 119 (1385)	**1.15 (1.10 to 1.20**)	**<0.001**
HbA1c (per 1 SD)	147 119 (1385)	**1.21 (1.16 to 1.26**)	**<0.001**

Analyses are adjusted for age, educational level and history of hormone replacement therapy. Statistically significant associations using a two-sided p<0.05 are presented in bold.

* Compared with postmenopausal women without surgical interference prior to menopause.

HbA1c, haemoglobin A1c.

Among women who had undergone natural menopause, lifetime HRT was associated with increased dementia risk (HR=1.13 (95% CI 1.00 to 1.27); table 3). No other associations between lifetime HRT and dementia risk were found. The number of incident dementia cases in the subgroups of bilateral oophorectomy and hysterectomy for HRT initiation was too small to analyse (no incident dementia case among those who had undergone hysterectomy and one incident dementia case among those who had undergone bilateral oophorectomy and initiated HRT more distant to menopause). No association between HRT initiation and dementia risk was found in the total study population or among participants who had undergone natural menopause (table 3).

**Table 3:** Associations of hormone replacement therapy with dementia risk

	Total study population		Natural menopause		Bilateral oophorectomy (with or without a hysterectomy)		Hysterectomy (without bilateral oophorectomy)	
	n=(cases)	Incident dementia HR (95% CI)	n=(cases)	Incident dementia HR (95% CI)	n=(cases)	Incident dementia HR (95% CI)	n=(cases)	Incident dementia HR (95% CI)
Lifetime HRT	147 119 (1385)	1.11 (0.99 to 1.23)	87 098 (1150)	**1.13 (1.00 to 1.27**)	9819 (109)	0.87 (0.51 to 1.48)	3891 (75)	1.05 (0.63 to 1.76)
Initiation HRT close to menopause*	35 836 (542)	1.04 (0.57 to 1.89)	31 866 (480)	0.78 (0.42 to 1.47)	1921 (23)	n/a†	2049 (39)	n/a†

Analyses are adjusted for age and educational level. Statistically significant associations using a two-sided p<0.05 are presented in bold.

* Initiation <61 years or initiation <11 years from menopause for women with menopause at age >44 years or initiation <52 years for women with menopause at age <45 years.

† No dementia case with hysterectomy and one dementia case with bilateral oophorectomy distant to menopause.

HRT, hormone replacement therapy.

Both fasting plasma glucose and HbA1c were associated with increased dementia risk (HR=1.15 (95% CI 1.10 to 1.20 per 1 SD) and HR=1.21 (95% CI 1.16 to 1.26) per 1 SD, respectively; table 2).

### Additional analyses on menopausal factors and dementia risk

Several sensitivity analyses were performed. Additional adjustments for cardiometabolic factors, health behavioural factors and deprivation did not change the association of age at natural menopause with decreased dementia risk (online supplemental eTable 3). However, the associations of early natural menopause and lifetime HRT among those with natural menopause with increased dementia risk attenuated and became statistically not significant after adjustment for cardiometabolic factors (HR=1.21 (95% CI 0.98 to 1.50) and HR=1.10 (95% CI 0.98 to 1.25), respectively; online supplemental eTable 3). This attenuation was the result of some selection bias in the study population with available cardiometabolic factors, as the increased dementia risk was attenuated in the population with data on cardiometabolic factors (HR=1.25 (95% CI 1.01 to 1.54)), and the increased dementia risk in those on lipid-modifying medications (HR=1.63 (95% CI 1.40 to 1.88)), as all other cardiometabolic factors were not associated with dementia risk. The associations between menopausal factors and dementia risk did not change after additional adjustment for polygenic risk scores for AD and no interactions of menopausal factors with polygenic risk scores on dementia risk were found (online supplemental eTables 4 and 5).

### Associations of menopausal factors with glycaemia

Associations of menopausal factors with markers of glycaemia are shown in online supplemental eTables 6 and 7. Bilateral oophorectomy (for those aged <50 years and ≥50 years at baseline) was associated with higher levels of fasting plasma glucose and HbA1c. Hysterectomy was associated with higher HbA1c levels (for those aged <50 years and ≥50 years at baseline) but only with fasting plasma glucose for those aged ≥50 years at baseline. No association was found between hysterectomy and fasting plasma glucose for those who underwent hysterectomy and were aged <50 years at baseline (online supplemental eTable 6).

Older age at natural menopause, bilateral oophorectomy and hysterectomy were associated with lower fasting plasma glucose and HbA1c levels. Furthermore, participants who underwent early natural menopause, early bilateral oophorectomy and early hysterectomy had higher levels of fasting plasma glucose and HbA1c (online supplemental eTable 6).

Lifetime HRT was associated with lower levels of fasting plasma glucose and HbA1c among women who had undergone natural menopause and bilateral oophorectomy, while no associations between lifetime HRT and glycaemia were found among those who had undergone hysterectomy (online supplemental eTable 7). No association between initiation of HRT and glycaemia was found (online supplemental eTable 7).

### Additional analyses on menopausal factors with glycaemia

The associations of bilateral oophorectomy, hysterectomy and age at natural menopause, age at bilateral oophorectomy and age at hysterectomy with glycaemia attenuated after additional adjustment for cardiometabolic factors (mainly because of waist circumference and lipid profile) and most became statistically non-significant (online supplemental eTable 8). The association of older age at bilateral oophorectomy with lower fasting plasma glucose remained statistically significant after full adjustment for cardiometabolic factors, health behavioural factors and deprivation, while the association of older age at hysterectomy with lower fasting plasma glucose became statistically non-significant after adjustment for cardiometabolic and health behavioural factors (online supplemental eTable 8). Older age at natural menopause remained statistically significantly associated with lower HbA1c levels after adjustment for cardiometabolic factors but became statistically non-significant after further adjustments for health behavioural factors (online supplemental eTable 8). Furthermore, bilateral oophorectomy for those aged <50 years at baseline and hysterectomy for those aged ≥50 years at baseline remained statistically significantly associated with higher HbA1c after full adjustments for cardiometabolic factors, health behavioural factors and deprivation.

Associations between lifetime HRT and lower glycaemia remained statistically significant after full adjustment for cardiometabolic factors, health behavioural factors and deprivation (online supplemental eTable 9).

### Mediation analysis

Of the menopausal factors, only age at natural menopause and lifetime HRT among those with natural menopause were associated with dementia risk. Therefore, mediation analyses were only performed to decompose the total effect of these factors on dementia risk into a direct and indirect effect via fasting plasma glucose and HbA1c. Table 4 shows that a small, but statistically significant part (1.7%) of the association between standardised age at natural menopause and decreased dementia risk (total effect=B=−0.00378 (95% CI −00527 to −0.00227)) is mediated by HbA1c levels (indirect effect=B=−0.00006 (95% CI −0.00010 to −0.000003)). The direct effect of standardised age at natural menopause on decreased dementia risk was almost equal to the total effect (direct effect=B=−0.00371 (95% CI −0.00523 to −0.00227), 98.3%). Furthermore, 4.7% of the association between early natural menopause and increased dementia risk (total effect=B=0.00375 (95% CI 0.00071 to 0.00678)) is mediated by HbA1c levels (indirect effect=B=0.00018 (95% CI 0.00009 to 0.00026)), while 95.3% could be explained by the direct effect of early natural menopause on increased dementia risk effect (direct effect=B=0.00357 (95% CI 0.00056 to 0.00658)). No mediation by fasting plasma glucose levels was found on the associations between standardised age at natural menopause and decreased dementia risk (indirect effect=B=−0.00002 (95% CI −0.00004 to 0.00000)) and early natural menopause and increased dementia risk (indirect effect=B=0.00006 (95% CI 0.00000 to 0.00011); table 4).

**Table 4:** Decomposed associations of age at natural menopause with incident dementia via glycaemia

	Number of participants in analysis (number of dementia cases)	Incident dementia B (95% CI)	Proportion mediated
Model			
Standardised age at natural menopause (SD 1 vs SD −1)*	82 426 (1035)		
Direct		**−0.00377 (−0.00529 to −0.00226**)	–
Indirect via fasting plasma glucose		−0.00002 (−0.00004 to 0.00000)	0.005 (−0.001 to 0.011)
Total		**−0.00379 (−0.00530 to −0.00227**)	–
Standardised age at natural menopause (SD 1 vs SD −1)*	82 426 (1035)		
Direct		**−0.00371 (−0.00523 to −0.00227**)	–
Indirect via HbA1c		**−0.00006 (−0.00010 to −0.00003**)	**0.017 (0.005 to 0.028**)
Total		**−0.00378 (−0.00527 to −0.00227**)	–
Early natural menopause	82 426 (1035)		
Direct		**0.00375 (0.00071 to 0.00678**)	–
Indirect via fasting plasma glucose		**0.00006 (0.00000 to 0.00011**)	0.016 (−0.001 to 0.033)
Total		**0.00381 (0.00076 to 0.00685**)	–
Early natural menopause	82 426 (1035)		
Direct		**0.00357 (0.00056 to 0.00658**)	–
Indirect via HbA1c		**0.00018 (0.00009 to 0.00026**)	**0.047 (0.009 to 0.085**)
Total		**0.00375 (0.00071 to 0.00678**)	–
Lifetime HRT among those with natural menopause	87 098 (1150)		
Direct		**0.00171 (0.00020 to 0.00323**)	–
Indirect via fasting plasma glucose		**−0.00008 (−0.00012 to −0.00004**)	
Total		**0.00163 (0.00012 to 0.00314**)	–
Lifetime HRT among those with natural menopause	87 098 (1150)		
Direct		**0.00182 (0.00030 to 0.00334**)	–
Indirect via HbA1c		**−0.00017 (−0.00022 to −0.00011**)	
Total		**0.00165 (0.00015 to 0.00361**)	–

Regression results are decomposed in direct, indirect and total effects and presented as B with 95% CI and adjusted for age, educational level and history of hormone replacement therapy. Statistically significant associations using a two-sided p<0.05 are presented in bold.

* Age is standardised and an SD of 1 is compared with an SD of −1.

HbA1c, haemoglobin A1c; HRT, hormone replacement.

A negative indirect effect of lifetime HRT on dementia risk via fasting plasma glucose and HbA1c levels was found (B=−0.00008 (95% CI −0.00012 to −0.00004) and B=−0.00017 (95% CI −0.00022 to −0.00011), respectively), while a positive direct effect of lifetime HRT on dementia risk was found (B=0.00171 (95% CI 0.00020 to 0.00323) and B=0.00182 (95% CI 0.00030 to 0.00334), table 4).

### Additional mediation analysis

Additional adjustments for cardiometabolic factors removed the significant indirect effect of older standardised age at natural menopause and early menopause via HbA1c on dementia risk (online supplemental eTable 10).

## Discussion

This study investigated associations of bilateral oophorectomy, hysterectomy, age at natural menopause and surgery, and lifetime and initiation of HRT with fasting plasma glucose and HbA1c levels and dementia risk. In line with our first hypothesis, direct effects of bilateral oophorectomy, hysterectomy and age at natural menopause or surgery on fasting plasma glucose and HbA1c levels were found. These findings corroborate previous data from meta-analyses linking these menopausal factors to T2DM^21 22^ and with findings from smaller studies that have linked surgical menopause and age at menopause to higher levels of glycaemia.^39^,^41^ However, only age at natural menopause was associated with increased dementia risk. This association was independent of genetic risk for dementia and did not differ between those with a low and high genetic predisposition for dementia. The absence of an association of bilateral oophorectomy and hysterectomy with elevated dementia risk is in line with a meta-analysis^8^ and with some work from UK Biobank,^7^ but not all.^6^ Differences in the analytical approach may have contributed to these discrepancies, specifically our approach to (a) account for competing risk of death or drop-out and (b) only considered likely postmenopausal women aged ≥50 years at baseline as reference group for clearer effect estimation.

The findings that older age at natural menopause is associated with a decreased risk and early natural menopause with an increased risk for dementia are similar to previous work using UK Biobank.^6 7^ A systematic review and meta-analysis coming to different conclusions included only one study with a focus on natural menopause,^9^ which was characterised by a small sample (n=592) and short follow-up (6.6 years).^42^ We did not find associations between age at bilateral oophorectomy and age at hysterectomy with dementia risk, which agrees with two meta-analyses that did not find associations of age at menopause and age at surgical menopause with dementia risk.^8 9^ Also, a younger age at hysterectomy was associated with increased dementia risk, whereas the relationship for age at bilateral oophorectomy and dementia risk appeared to be U-shaped using UK Biobank data.^7^ The use of restricted cubic spline plots might explain these differences in results, but the rationale for a later age at surgical menopause increasing dementia risk is unclear. Although early age at surgical menopause, defined as <45 years, was associated with increased dementia risk,^8^ the one out of the two meta-analysed studies with clinical dementia ascertainment did not find an association between age at surgical menopause and dementia risk,^43^ corroborating our results. In conclusion, studies that have investigated the associations of age at natural menopause, bilateral oophorectomy and hysterectomy and differentiated between the type of menopause with clinically ascertained incident dementia remain scarce. Therefore, replication of our findings in large long-term follow-up cohorts is recommended.

In line with our second hypothesis, lifetime HRT was associated with lower levels of glycaemia among those who had undergone natural menopause and bilateral oophorectomy. However, no association between initiation of HRT and glycaemia was found. As expected, the associations between lifetime HRT and dementia risk (independent of polygenic genetic risk) differed depending on age and menopausal type,^11^ while no association between HRT initiation and dementia risk was found. The increased dementia risk for lifetime HRT among women who have undergone natural menopause corroborates recent findings from other large population-based cohorts.^12^,^16^ Although these results support that lifetime HRT might be associated with both glycaemia levels and dementia risk, no support for the ‘timing hypothesis’ was found.^30 44^ This might be because of the relative short follow-up duration, which resulted in a lack of dementia cases in subgroup analyses. As said, replication of our findings in long-term followed-up cohorts is recommended. In addition, statistical power might be further increased by cohorts focused on surgical menopause. However, caution is needed in the interpretation of these results, as the association between HRT and the brain is complex and involves many interconnected factors. Additionally, women reporting more contained use of HRT may have also been treated under new protocols of shorter duration of HRT with more targeted, safer drugs. Updated findings of the KEEPS study have shown that there are no long-term cognitive effects of short-term exposure to HRT started in early menopause versus placebo providing reassurance about the neurocognitive safety of HRT during the menopausal transition.^45^

In line with our third hypothesis, HbA1c level marginally mediated the increased risk for dementia in those with an older age of natural menopause (1.7%) and those with early natural menopause (4.7%). This finding provides additional evidence that hyperglycaemia may be involved in increased dementia risk in women with an earlier onset of natural menopause. Although the mediated part of the association between age at natural menopause and dementia risk was small, hyperglycaemia might be an important target to prevent or delay onset of dementia in women with an early age of natural menopause. Lifetime HRT showed direct positive effects on dementia risk but indirect negative effects via fasting plasma glucose and HbA1c levels. This suggests the presence of further unobserved determinants of HRT and/or risk of dementia, and further unobserved mediators to investigate in future studies.

Results of this study contribute to recommendations for clinical practice and further research. Women who undergo natural menopause at a young age should be monitored for both hyperglycaemia and dementia. This might even be more beneficial for the prevention and delay of cognitive decline, as convincing evidence has linked an earlier age of natural menopause and surgical menopause to worse cognitive performance in later life and more rapid cognitive decline.^8 9^ In addition, all women undergoing bilateral oophorectomy, hysterectomy and especially those at a younger age should be followed up to monitor their levels of glycaemia. Sensitivity analyses showed that the associations between menopausal factors and higher levels of glycaemia attenuated after adjustment for other metabolic risk factors. Therefore, it is recommended to consider the whole cardiometabolic risk profile, to not only prevent the development of hyperglycaemia^46^ but also reduce dementia risk.^47^

Clinical recommendations about the use of HRT in dementia prevention are complex and require attention to variability between women.^48^ The North American Menopause Society recommends HRT to prevent dementia for women who undergo menopause in the normal age range (45–54 years) and experience vasomotor symptoms.^30^ However, no recommendations are made for other groups. Future research should differentiate types of HRT and investigate the role of HRT on glycaemia and dementia risk in subgroups differentiated by (1) sociodemographic profiles, (2) premature, early, normal age and late menopause, (2) natural and surgical menopause, including hysterectomy with ovarian conservation and (3) vasomotor symptoms.^11^ A large part of the association between age at natural menopause and dementia risk remained unexplained in our study. Natural menopause depends on multiple factors such as genetics, ethnicity, environment, inflammation, lifestyle, socioeconomic position and comorbidities.^49^,^52^ All these factors have also been linked to dementia risk.^53^,^55^ Furthermore, later age at natural menopause has been related to increased longevity and better health outcomes^56^,^58^ that may reflect some innate resilience or protectiveness that delays biological ageing. Therefore, further investigations of determinants and potential mechanisms underlying the association between age at natural menopause and dementia risk are needed.

Strengths of this study are the large sample size and long follow-up duration that included hospital records, which enabled investigation of bilateral oophorectomy and hysterectomy in the general population and incident dementia. Furthermore, we performed a range of sensitivity analyses to evaluate effects of potential confounders and included two measures of glycaemia. There are also some limitations. First, the mean follow-up period of 12.5 years might have been still short to assess incident dementia in this relatively young study population. Although we found some associations with incident dementia, the relatively small incidence of dementia (0.9%) suggests some underdiagnosis. Second, menopausal factors were self-reported. Misclassification bias in which participants are falsely categorised as premenopausal or postmenopausal might have affected our results. Third, the average age of natural menopause in our study sample was 50.3 years, which is earlier than the national average of 51 years in the UK.^59^ Although the difference is small, UK Biobank might not be completely representative of the general population. However, valid assessment of exposure-disease relationships may be generalisable even if participants are not fully representative of the population.^60^ Fourth, women who underwent bilateral oophorectomy and hysterectomy might have been better monitored than those who were premenopausal or underwent natural menopause. Indeed, women who underwent bilateral oophorectomy or hysterectomy were more likely to use lipid-modifying or antihypertensive medication, possibly lowering their dementia risk. In addition, women who had undergone bilateral oophorectomy more often used HRT in line with postsurgery protocol. Fifth, the inability to distinguish between types of dementia and possible heterogeneity in underlying pathologies may have affected our results. The contribution of glycaemia to the association between early age at natural menopause and incident dementia may be higher for vascular dementia^61 62^ and/or AD caused by insulin resistance, also called ‘type 3 diabetes’.^63^ Sixth, there might have been reverse causation in which higher levels of glycaemia affect the age of natural menopause, as T2DM may accelerate the onset of menopause.^64^ Cardiometabolic and behavioural mediators may have also affected the age of natural menopause.^65 66^ Studies like the Study of Women’s Health Across the Nation collecting fine-grained data of women going through menopause may provide better insight into the temporality of age at menopause and metabolic profile changes. Seventh, no information on the type of HRT was available. Lastly, no data on vasomotor symptoms was collected. HRT may be beneficial for women who undergo menopause at a normal age with vasomotor symptoms, while its effect on the brain regardless of vasomotor symptoms could not be confirmed.^11^

In conclusion, this study observed associations between bilateral oophorectomy, hysterectomy and earlier age at natural menopause and surgery with higher levels of glycaemia, while lifetime HRT was associated with lower levels of glycaemia. HbA1c levels marginally mediated increased dementia risk in those with an earlier age of natural menopause. The metabolic profile of women undergoing natural menopause at an early age should be monitored to not only prevent hyperglycaemia but also decrease dementia risk. Studies investigating alternative mechanisms may further elucidate the complex associations between menopausal factors, HRT and dementia risk.

## Supplementary material

10.1136/bmjph-2024-002120 online supplemental file 1

## Data Availability

Data are available on reasonable request.

## References

[1] Huque H, Eramudugolla R, Chidiac B (2023). Could Country-Level Factors Explain Sex Differences in Dementia Incidence and Prevalence? A Systematic Review and Meta-Analysis. J Alzheimers Dis.

[2] Vest RS, Pike CJ (2013). Gender, sex steroid hormones, and Alzheimer’s disease. Horm Behav.

[3] Maki PM, Thurston RC (2020). Menopause and Brain Health: Hormonal Changes Are Only Part of the Story. Front Neurol.

[4] Utian WH (2004). Menopause-related definitions. Int Congr Ser.

[5] NHS (2023). Early menopause: national health service of the United Kingdom; 2021. https://www.nhs.uk/conditions/early-menopause/#:~:text=Early%20menopause%20happens%20when%20a,ages%20of%2045%20and%2055.

[6] Costantino M, Pigeau G, Parent O Menopause, brain anatomy, cognition and Alzheimer's Disease. bioRxiv.

[7] Gong J, Harris K, Peters SAE (2022). Reproductive factors and the risk of incident dementia: A cohort study of UK Biobank participants. PLoS Med.

[8] Georgakis MK, Beskou-Kontou T, Theodoridis I (2019). Surgical menopause in association with cognitive function and risk of dementia: A systematic review and meta-analysis. Psychoneuroendocrinology.

[9] Georgakis MK, Kalogirou EI, Diamantaras A-A (2016). Age at menopause and duration of reproductive period in association with dementia and cognitive function: A systematic review and meta-analysis. Psychoneuroendocrinology.

[10] Rocca WA, Grossardt BR, Shuster LT (2011). Oophorectomy, menopause, estrogen treatment, and cognitive aging: clinical evidence for a window of opportunity. Brain Res.

[11] Rocca WA, Kantarci K, Faubion SS (2024). Risks and benefits of hormone therapy after menopause for cognitive decline and dementia: A conceptual review. Maturitas.

[12] Savolainen-Peltonen H, Rahkola-Soisalo P, Hoti F (2019). Use of postmenopausal hormone therapy and risk of Alzheimer’s disease in Finland: nationwide case-control study. BMJ.

[13] Vinogradova Y, Dening T, Hippisley-Cox J (2021). Use of menopausal hormone therapy and risk of dementia: nested case-control studies using QResearch and CPRD databases. BMJ.

[14] Sung Y-F, Tsai C-T, Kuo C-Y (2022). Use of Hormone Replacement Therapy and Risk of Dementia: A Nationwide Cohort Study. Neurology (ECronicon).

[15] Pourhadi N, Mørch LS, Holm EA (2023). Menopausal hormone therapy and dementia: nationwide, nested case-control study. BMJ.

[16] Løkkegaard LE, Thinggaard M, Nygaard M (2022). Systemic hormone therapy and dementia: A nested case-control and co-twin control study. Maturitas.

[17] Kanaya AM, Herrington D, Vittinghoff E (2003). Glycemic effects of postmenopausal hormone therapy: the Heart and Estrogen/progestin Replacement Study. A randomized, double-blind, placebo-controlled trial. Ann Intern Med.

[18] Crespo CJ, Smit E, Snelling A (2002). Hormone replacement therapy and its relationship to lipid and glucose metabolism in diabetic and nondiabetic postmenopausal women: results from the Third National Health and Nutrition Examination Survey (NHANES III). Diabetes Care.

[19] Slopien R, Wender-Ozegowska E, Rogowicz-Frontczak A (2018). Menopause and diabetes: EMAS clinical guide. Maturitas.

[20] van der Kooij MA (2020). The impact of chronic stress on energy metabolism. Mol Cell Neurosci.

[21] Guo C, Li Q, Tian G (2019). Association of age at menopause and type 2 diabetes: A systematic review and dose-response meta-analysis of cohort studies. Prim Care Diabetes.

[22] Chiang C-H, Chen W, Tsai I-J (2021). Diabetes mellitus risk after hysterectomy: A population-based retrospective cohort study. Medicine (Baltimore).

[23] Wang M, Gan W, Kartsonaki C (2022). Menopausal status, age at natural menopause and risk of diabetes in China: a 10-year prospective study of 300,000 women. *Nutr Metab (Lond*).

[24] WHO (2022). Menopause.

[25] Kapogiannis D, Mattson MP (2011). Disrupted energy metabolism and neuronal circuit dysfunction in cognitive impairment and Alzheimer’s disease. Lancet Neurol.

[26] Biessels GJ, Staekenborg S, Brunner E (2006). Risk of dementia in diabetes mellitus: a systematic review. Lancet Neurol.

[27] Crane PK, Walker R, Hubbard RA (2013). Glucose levels and risk of dementia. N Engl J Med.

[28] Roglic G (2016). WHO Global report on diabetes: A summary. *Int J Non-Commun Dis*.

[29] Sudlow C, Gallacher J, Allen N (2015). UK biobank: an open access resource for identifying the causes of a wide range of complex diseases of middle and old age. PLoS Med.

[30] Faubion SS, Crandall CJ, Davis L (2022). The 2022 hormone therapy position statement of The North American Menopause Society. Menopause.

[31] Wilkinson T, Schnier C, Bush K (2019). Identifying dementia outcomes in UK Biobank: a validation study of primary care, hospital admissions and mortality data. Eur J Epidemiol.

[32] Sommerlad A, Perera G, Singh‐Manoux A (2018). Accuracy of general hospital dementia diagnoses in England: Sensitivity, specificity, and predictors of diagnostic accuracy 2008–2016. Alzheimers Dement.

[33] Liu J, Jin X, Liu W (2023). The risk of long-term cardiometabolic disease in women with premature or early menopause: A systematic review and meta-analysis. Front Cardiovasc Med.

[34] Nappi RE, Chedraui P, Lambrinoudaki I (2022). Menopause: a cardiometabolic transition. Lancet Diabetes Endocrinol.

[35] Farahmand M, Ramezani Tehrani F, Bahri Khomami M (2015). Surgical menopause versus natural menopause and cardio-metabolic disturbances: A 12-year population-based cohort study. J Endocrinol Invest.

[36] Biobank TU (2007). UK biobank: protocol for a large-scale prospective epidemiological resource. http://www.ukbiobank.ac.uk/wp-content/uploads/2011/11/UK-Biobank-Protocol.pdf.

[37] Townsend P (1987). Deprivation. J Soc Pol.

[38] Lourida I, Hannon E, Littlejohns TJ (2019). Association of Lifestyle and Genetic Risk With Incidence of Dementia. JAMA.

[39] O’Keeffe LM, Kuh D, Fraser A (2020). Age at period cessation and trajectories of cardiovascular risk factors across mid and later life. Heart.

[40] Kalyan S, Hitchcock CL, Pudek M (2011). Acute Effects of Premenopausal Hysterectomy with Bilateral Oophorectomy on Serum Lipids, Hormonal Values, Inflammatory Markers, and Metabolism. J Gynecol Surg.

[41] Kuh D, Langenberg C, Hardy R (2005). Cardiovascular risk at age 53 years in relation to the menopause transition and use of hormone replacement therapy: a prospective British birth cohort study. *BJOG*.

[42] Bove R, Secor E, Chibnik LB (2014). Age at surgical menopause influences cognitive decline and Alzheimer pathology in older women. Neurology (ECronicon).

[43] Rocca WA, Gazzuola-Rocca L, Smith CY (2016). Accelerated Accumulation of Multimorbidity After Bilateral Oophorectomy: A Population-Based Cohort Study. Mayo Clin Proc.

[44] Clarkson TB, Meléndez GC, Appt SE (2013). Timing hypothesis for postmenopausal hormone therapy: its origin, current status, and future. Menopause.

[45] Gleason CE, Dowling NM, Kara F (2024). Long-term cognitive effects of menopausal hormone therapy: Findings from the KEEPS Continuation Study. PLoS Med.

[46] Law TK, Yan AT, Gupta A (2015). Primary prevention of cardiovascular disease: global cardiovascular risk assessment and management in clinical practice. Eur Heart J Qual Care Clin Outcomes.

[47] Stefanowski B, Kucharski M, Szeliga A (2023). Cognitive decline and dementia in women after menopause: Prevention strategies. Maturitas.

[48] Edwards H, Duchesne A, Au AS (2019). The many menopauses: searching the cognitive research literature for menopause types. *Menopause*.

[49] Gold EB, Crawford SL, Avis NE (2013). Factors related to age at natural menopause: longitudinal analyses from SWAN. Am J Epidemiol.

[50] Voorhuis M, Onland-Moret NC, van der Schouw YT (2010). Human studies on genetics of the age at natural menopause: a systematic review. Hum Reprod Update.

[51] Christensen A, Pike CJ (2015). Menopause, obesity and inflammation: interactive risk factors for Alzheimer’s disease. Front Aging Neurosci.

[52] Roa-Díaz ZM, Raguindin PF, Bano A (2021). Menopause and cardiometabolic diseases: What we (don’t) know and why it matters. Maturitas.

[53] Livingston G, Huntley J, Sommerlad A (2020). Dementia prevention, intervention, and care: 2020 report of the Lancet Commission. Lancet.

[54] Loy CT, Schofield PR, Turner AM (2014). Genetics of dementia. The Lancet.

[55] Simone MJ, Tan ZS (2011). The Role of Inflammation in the Pathogenesis of Delirium and Dementia in Older Adults: A Review. CNS Neurosci Ther.

[56] Muka T, Oliver-Williams C, Kunutsor S (2016). Association of Age at Onset of Menopause and Time Since Onset of Menopause With Cardiovascular Outcomes, Intermediate Vascular Traits, and All-Cause Mortality. JAMA Cardiol.

[57] Gagnon A (2015). Natural fertility and longevity. Fertil Steril.

[58] Faubion SS, Kuhle CL, Shuster LT (2015). Long-term health consequences of premature or early menopause and considerations for management. Climacteric.

[59] Sarri G, Davies M, Lumsden MA (2015). Diagnosis and management of menopause: summary of NICE guidance. BMJ.

[60] Fry A, Littlejohns TJ, Sudlow C (2017). Comparison of Sociodemographic and Health-Related Characteristics of UK Biobank Participants With Those of the General Population. Am J Epidemiol.

[61] Ahtiluoto S, Polvikoski T, Peltonen M (2010). Diabetes, Alzheimer disease, and vascular dementia: a population-based neuropathologic study. Neurology (ECronicon).

[62] Javanshiri K, Waldö ML, Friberg N (2018). Atherosclerosis, Hypertension, and Diabetes in Alzheimer’s Disease, Vascular Dementia, and Mixed Dementia: Prevalence and Presentation. J Alzheimers Dis.

[63] de la Monte SM, Wands JR (2008). Alzheimer’s Disease is Type 3 Diabetes—Evidence Reviewed. J Diabetes Sci Technol.

[64] Brand JS, Onland-Moret NC, Eijkemans MJC (2015). Diabetes and onset of natural menopause: results from the European Prospective Investigation into Cancer and Nutrition. *Hum Reprod*.

[65] Peycheva D, Sullivan A, Hardy R (2022). Risk factors for natural menopause before the age of 45: evidence from two British population-based birth cohort studies. BMC Womens Health.

[66] Mishra GD, Chung H-F, Cano A (2019). EMAS position statement: Predictors of premature and early natural menopause. Maturitas.

