# Supplementary material for: Associations of age at menopause, bilateral oophorectomy, hysterectomy and hormone replacement therapy with glycaemia and risk of dementia: a study based on the population-based UK Biobank cohort
Source: BMJ Public Health. 2025 Jul 27;3(2):e002120. doi: 10.1136/bmjph-2024-002120 (PMC12306347; doi:10.1136/bmjph-2024-002120)
Supplement: online supplemental file 1 [file bmjph-3-2-s001.pdf]

Supplemental Material

Associations of age at menopause, bilateral oophorectomy, hysterectomy, and hormone replacement therapy with glycemia and risk of dementia: a study based on the population-based UK Biobank cohort

Anouk FJ Geraets, Ph.D.[1]*, Katherine J Ford, Ph.D.[2], Patrick May, Ph.D.[3], Emma J Kidd, Ph.D.[4] & Anja K Leist, Ph.D.[1]

[1] Department of Social Sciences, University of Luxembourg, Esch-sur-Alzette, Luxembourg, [2] Department of Psychology, Carleton University, Ottawa, Canada, [3] Luxembourg Centre for Systems Biomedicine, University of Luxembourg, Esch-sur-Alzette, Luxembourg, [4] School of Pharmacy and Pharmaceutical Sciences, Cardiff University, Cardiff, United Kingdom

Supplemental methods

Menopausal factors

Baseline data on menopausal status, bilateral oophorectomy, and hysterectomy were self-reported (see Supplemental Material for exact wording). with the questions, "Have you had your menopause (periods stopped)?", "Have you had BOTH ovaries removed?", and "Have you had a hysterectomy (womb removed)?", respectively. Age at natural menopause was assessed with the question "How old were you when your periods stopped?" and age at surgical menopause with the questions "How old were you when you had BOTH ovaries removed?" and "How old were you when you had your hysterectomy?".

Supplemental tables

eTable 1. Characteristics of female participants included and excluded in analyses

Characteristic	Included (n=147,119)	Excluded (n=126,182)	*p*-value
**Demographics**			
Age (years)	55.2 ± 8.0	57.7 ± 7.7	<0.001
High educational attainment, n (%)	61,055 (41.5)	23,445 (39.6)	<0.001
Townsend deprivation index [a]	2.08 ± 0.33	2.13 ± 0.34	<0.001
**Dementia**			
Incident dementia, n (%)	1,385 (0.9)	2,249 (1.8)	<0.001
**Menopausal factors**			
Pre-menopausal, n (%)	46,311 (31.5)	17,143 (18.4)	<0.001
Natural menopause, n (%)	87,098 (59.2)	64,151 (68.7)	<0.001
Bilateral oophorectomy, n (%)	9,819 (6.7)	8,098 (8.7)	<0.001
Hysterectomy, n (%)	3,816 (2.6)	75 (5.4)	<0.001
Age natural menopause (years)	50.3 ± 4.5	50.0 ± 4.8	<0.001
Early natural menopause (< 45 years), n (%)	7,699 (9.3)	6,631 (11.0)	<0.001
Age bilateral oophorectomy (years)	45.8 ± 6.7	45.9 ± 7.1	0.263
Early bilateral oophorectomy (< 45 years), n (%)	3,535 (36.6)	2,842 (36.4)	0.807
Age hysterectomy (years)	41.5 ± 6.5	41.0 ± 6.8	0.008
Early hysterectomy (< 45 years), n (%)	2,633 (68.6)	2,667 (69.7)	0.287
Ever used HR, n (%)	49,844 (33.9)	54,058 (43.4)	<0.001
Age start HRT (years)	47.7 ± 5.3	47.1 ± 5.6	<0.001
Initiation HRT close to menopause [a], n (%)	35,372 (98.7)	28,378 (98.3)	<0.001
**Glycemia**			
Fasting plasma glucose (mmol/L)	5.0 ± 1.0	5.1 ± 1.2	<0.001
HbA1c (mmol/mol)	35.4 ± 5.6	36.4 ± 6.4	<0.001
**Cardiometabolic risk factors**			
Waist circumference (cm)	83.7 ± 12.3	86.0 ± 12.7	<0.001
Triglyceride-to-HDL ratio	1.03 ± 0.77	1.20 ± 0.88	<0.001
Lipid-modifying medication, n (%)	14,650 (10.0)	19,957 (15.8)	<0.001
Systolic blood pressure (mm Hg)	135.6 ± 20.0	139.2 ± 20.5	<0.001
Diastolic blood pressure (mm Hg)	80.4 ± 10.6	81.1 ± 10.6	<0.001
Antihypertensive medication, n (%)	13,243 (9.0)	14,675 (11.6)	<0.001
**Behavioral risk factors**			
Alcohol use frequency days per week (0/1-4/5-7), n (%)	49,887/70,970/26,184 (33.9/48.3/17.8)	52,784/55,067/17,670 (42.1/43.9/14.1)	<0.001
Current smoker, n (%)	11,628 (7.9)	12,732 (10.1)	<0.001
Moderate physical activity (min/day) [b]	3.7 ± 0.9	3.8 ± 0.9	<0.001
Ever had mental health problems, n (%)	58,701 (40.2)	52,125 (42.0)	<0.001
**Genetic risk**			
Standardized polygenic risk scores for dementia	0.05 (1.00)	0.04 (0.99)	0.0190

Data are presented as means ± standard deviation or number (%). HRT indicates hormone replacement therapy; HbA1c, hemoglobin A1c; HDL, high-density lipoprotein. [a] Initiation <61 years or initiation <11 years from menopause for women with menopause at age >44 years or initiation <52 years for women with menopause at age <45 years. [b] Values are log transformed because of skewed distribution.

eTable 2. Baseline characteristics of study population by menopausal group

Characteristic	Pre-menopausal	Natural menopause	Bilateral oophorectomy	Hysterectomy
	(n=46,311)	(n=87,098)	(n=9,919)	(n=3,891)
**Demographics**				
Age (years)	46.0 ± 3.9	59.5 ± 5.4	58.0 ± 6.6	60.5 ± 6.0
High educational attainment, n (%)	20,851 (45.0)	35,753 (41.1)	3,277 (33.4)	1,174 (30.2)
Townsend deprivation index [a]	2.11 ± 0.34	2.06 ± 0.32	2.07 ± 0.33	2.06 ± 0.33
**Dementia**				
Incident dementia, n (%)	51 (0.1)	1,150 (1.3)	109 (1.1)	75 (1.9)
**Menopausal factors**				
Ever used HR, n (%)	1,430 (3.1)	37,474 (43.0)	8,352 (85.1)	2,588 (6.5)
Age start HRT (years)	45.2 ± 6.1	48.5 ± 4.8	44.8 ± 5.8	46.1 ± 5.5
Initiation HRT close to menopause [a], n (%)	n/a	31,571 (99.1)	1,910 (99.4)	1,891 (92.3)
**Glycemia**				
Fasting plasma glucose (mmol/L)	4.9 ± 0.9	5.1 ± 1.0	5.1 ± 1.1	5.2 ± 1.2
HbA1c (mmol/mol)	33.4 ± 4.9	36.3 ± 5.5	36.3 ± 6.2	36.9 ± 6.8
**Cardiometabolic risk factors**				
Waist circumference (cm)	81.9 ± 12.2	84.1 ± 12.1	86.8 ± 12.8	86.3 ± 12.2
Triglyceride-to-HDL ratio	0.91 ± 0.71	1.06 ± 0.78	1.24 ± 0.91	1.22 ± 0.88
Lipid-modifying medication, n (%)	1,101 (2.4)	11,135 (12.8)	1,620 (16.5)	794 (20.4)
Systolic blood pressure (mm Hg)	127.5 ± 16.9	139.3 ± 20.1	139.4 ± 20.1	140.7 ± 20.2
Diastolic blood pressure (mm Hg)	78.9 ± 10.5	81.0 ± 10.5	81.6 ± 10.6	81.1 ± 10.5
Antihypertensive medication, n (%)	1,835 (4.0)	9,481 (10.9)	1,386 (14.1)	541 (13.9)
**Behavioral risk factors**				
Alcohol use frequency days per week (0/1-4/5-7), n (%)	15,607/24,130/6,545 (33.7/52.1/14.1)	28,869/40,730/17,456 (33.2/46.8/20.1)	3,879/4,391/1,547 (39.5/44.7/15.8)	636/1,719/1,532 (43.0/38.7/18.3)
Current smoker, n (%)	4,366 (9.4)	6,157 (7.1)	810 (8.3)	295 (7.6)
Moderate physical activity (min/day) [b]	3.6 ± 0.8	3.8 ± 0.9	3.8 ± 0.9	3.9 ± 0.9
Ever had mental health problems, n (%)	18,155 (39.5)	34,024 (39.3)	4,689 (48.1)	1,833 (47.4)
**Genetic risk**				
Standardized polygenic risk scores for dementia	0.05 (1.00)	0.05 (1.00)	0.06 (1.01)	0.05 (1.02)

Data are presented as means ± standard deviation or number (%). HRT indicates hormone replacement therapy; HbA1c, hemoglobin A1c; HDL, high-density lipoprotein. [a] Initiation <61 years or initiation <11 years from menopause for women with menopause at age >44 years or initiation <52 years for women with menopause at age <45 years. [b] Values are log transformed because of skewed distribution.

eTable 3. Additional analyses for the associations of menopausal factors with dementia risk

	**Number of participants in analysis (number of dementia cases)**	**Incident dementia HR (95% CI)**	***p*-value**
**Age natural menopause (years)**			
**Model 1**	82,426 (1,035)		
Age natural menopause (years)		**0.97 (0.96;0.98)**	**<0.001**
**Model 2: model 1 + cardiometabolic factors**	76,946 (950)		
Age natural menopause (years)		**0.97 (0.96;0.99)**	**<0.001**
**Model 3: model 2 + health behaviour factors**	58,432 (675)		
Age natural menopause (years)		**0.97 (0.96;0.99)**	**0.001**
**Model 4: model 3 + deprivation**	58,372 (675)		
Age natural menopause (years)		**0.97 (0.96;0.99)**	**0.001**
**Early natural menopause (<45 years)**			
**Model 1**	82,426 (1,035)		
Age natural menopause (years)		**1.31 (1.08;1.60)**	**0.007**
**Model 2: model 1 + cardiometabolic factors**	76,946 (950)		
Age natural menopause (years)		1.21 (0.98;1.50)	0.074
**Model 3: model 2 + health behaviour factors**	58,432 (675)		
Age natural menopause (years)		1.14 (0.88;1.47)	0.315
**Model 4: model 3 + deprivation**	58,372 (675)		
Age natural menopause (years)		1.13 (0.88;1.46)	0.340
**Lifetime HRT among those with natural menopause**			
**Model 1**	87,098 (1,150)		
Age natural menopause (years)		**1.13 (1.00;1.27)**	**0.043**
**Model 2: model 1 + cardiometabolic factors**	81,348 (1,055)		
Age natural menopause (years)		1.10 (0.98;1.25)	0.113
**Model 3: model 2 + health behaviour factors**	61,285 (741)		
Age natural menopause (years)		1.06 (0.92;1.23)	0.411
**Model 4: model 3 + deprivation**	61,224 (741)		
Age natural menopause (years)		1.06 (0.92;1.23)	0.411

HR indicates hazard ratio; CI, confidence interval, HRT, hormone replacement therapy. Statistically significant associations using a two-sided *p*-value < 0.05 are presented in bold. Model 1 is adjusted for age, educational level, and history of hormone replacement therapy; model 2 is additionally adjusted for waist circumference, triglyceride-to-high-density lipoprotein ratio, systolic blood pressure, use of cholesterol lowering medication, and blood pressure lowering medication; model 3 is additionally adjusted for alcohol use frequency, current tobacco use, moderate physical activity, and history of mental health problems; model 4 is additionally adjusted for deprivation.

eTable 4. Associations between menopausal factors and dementia risk additional adjusted for and interactions with polygenic risk

	Number of participants in analysis (number of dementia cases)	Incident dementia HR (95% CI)	*p*-value
**Additional adjustment for polygenic risk score**			
Bilateral oophorectomy aged ≥ 50 years[a]	94,854 (1,246)	0.84 (0.69;1.03)	0.103
Hysterectomy aged ≥ 50 years[a]	89,926 (1,214)	1.08 (0.85;1.37)	0.527
Age natural menopause (years)	81,699 (1,027)	**0.97 (0.96;0.98)**	**<0.001**
Early natural menopause	81,699 (1,027)	**1.31 (1.07;1.59)**	**0.008**
Age bilateral oophorectomy (years)	9,567 (101)	0.98 (0.95;1.00)	0.097
Early bilateral oophorectomy	9,567 (101)	1.35 (0.90;2.05)	0.150
Age hysterectomy (age)	3,798 (73)	0.98 (0.94;1.02)	0.265
Early hysterectomy	3,798 (73)	1.58 (0.93;2.68)	0.087
**Interactions with polygenic risk score**			
Bilateral oophorectomy aged ≥ 50 years[a] * polygenic risk score	94,854 (1,246)	1.12 (0.95;1.32)	0.172
Hysterectomy aged ≥ 50 years[a] * polygenic risk score	89,926 (1,214)	0.93 (0.76;1.14)	0.509
Age natural menopause (years) * polygenic risk score	81,699 (1,027)	1.01 (1.00;1.02)	0.161
Early natural menopause * polygenic risk score	81,699 (1,027)	0.92 (0.78;1.10)	0.364
Age bilateral oophorectomy (years) * polygenic risk score	9,567 (101)	1.02 (1.00;1.04)	0.053
Early bilateral oophorectomy * polygenic risk score	9,567 (101)	0.86 (0.62;1.20)	0.388
Age hysterectomy (age) * polygenic risk score	3,798 (73)	1.03 (1.00;1.06)	0.098
Early hysterectomy * polygenic risk score	3,798 (73)	0.71 (0.46;1.10)	0.126

HR indicates hazard ratio; CI, confidence interval; SD, standard deviation. Analyses are adjusted for age, educational level, and history of hormone replacement therapy. Statistically significant associations using a two-sided *p*-value < 0.05 are presented in bold. [a]Compared to post-menopausal women without surgical interference prior to menopause.

eTable 5. Associations between lifetime hormone replacement therapy and dementia risk additional adjustment for and interactions with polygenic risk

	**Total study population**		**Natural menopause**		**Bilateral oophorectomy (with or without a hysterectomy)**		**Hysterectomy (without bilateral oophorectomy)**	
	**n= (cases)**	**Incident dementia HR (95% CI)**	**n= (cases)**	**Incident dementia HR (95% CI)**	**n= (cases)**	**Incident dementia HR (95% CI)**	**n= (cases)**	**Incident dementia HR (95% CI)**
**Additional adjustment for polygenic risk score**								
Lifetime HRT	145,810 (1,374)	1.11 (0.99;1.23)	86,322 (1,142)	**1.14 (1.01;1.28)**	9,714 (109)	0.88 (0.52;1.49)	3,851 (74)	1.07 (0.63;1.81)
**Interactions with polygenic risk score**								
Lifetime HRT * polygenic risk score	145,810 (1,374)	1.09 (0.92;1.18)	86,322 (1,142)	1.08 (0.94;1.24)	9,714 (109)	0.83 (0.43;1.60)	3,851 (74)	1.06 (0.58;1.92)

HR indicates hazard ratio; CI, confidence interval; SD, standard deviation; HRT, hormone replacement therapy. Analyses are adjusted for age and educational level. Statistically significant associations using a two-sided *p*-value < 0.05 are presented in bold.

eTable 6. Associations of menopausal factors with markers of glycemia

Menopausal factors	Number of participants in analysis	Fasting plasma glucose (per 1 SD) B (95% CI)	HbA1c (per 1 SD) B (95% CI)
Bilateral oophorectomy aged < 50 years [a]	47,518	**0.052 (0.003;0.102)**	**0.217 (0.166;0.267)**
Bilateral oophorectomy aged ≥ 50 years [b]	95,711	**0.064 (0.044;0.083)**	**0.061 (0.042;0.080)**
Hysterectomy aged < 50 years [a]	46,561	-0.002 (-0.091;0.087)	**0.140 (0.048;0.231)**
Hysterectomy aged ≥ 50 years [b]	90,739	**0.065 (0.037;0.093)**	**0.087 (0.059;0.114)**
Age natural menopause (years)	82,426	**-0.001 (-0.003;-0.000)**	**-0.003 (-0.005;-0.002)**
Early natural menopause	82,426	**0.028 (0.008;0.048)**	**0.064 (0.045;0.083)**
Age bilateral oophorectomy (years)	9,667	**-0.008 (-0.011;-0.005)**	**-0.006 (-0.009;-0.003)**
Early bilateral oophorectomy	9,667	**0.087 (0.046;0.127)**	**0.084 (0.043;0.124)**
Age hysterectomy (age)	3,837	**-0.009 (-0.014;-0.003)**	**-0.009 (-0.014;-0.004)**
Early hysterectomy	3,837	**0.076 (0.007;0.146)**	**0.079 (0.009;0.150)**

B indicates unstandardized regression coefficient; CI, confidence interval; SD, standard deviation; HbA1c, hemoglobin A1c. Analyses are adjusted for age, educational level, and history of hormone replacement therapy. Statistically significant associations using a two-sided *p*-value < 0.05 are presented in bold. [a] Compared to pre-menopausal women without surgical interference. [b] Compared to post-menopausal women without surgical interference prior to menopause.

eTable 7. Associations of hormone replacement therapy with markers of glycemia

	**Total study population**			**Natural menopause**			**Bilateral oophorectomy (with or without a hysterectomy)**			**Hysterectomy (without bilateral oophorectomy)**		
	**n= (cases)**	**Fasting plasma glucose (per 1 SD)**<br>**B (95% CI)**	**HbA1c (per 1 SD)**<br>**B (95% CI)**	**n= (cases)**	**Fasting plasma glucose (per 1 SD)**<br>**B (95% CI)**	**HbA1c (per 1 SD)**<br>**B (95% CI)**	**n= (cases)**	**Fasting plasma glucose (per 1 SD)**<br>**B (95% CI)**	**HbA1c (per 1 SD)**<br>**B (95% CI)**	**n= (cases)**	**Fasting plasma glucose (per 1 SD)**<br>**B (95% CI)**	**HbA1c (per 1 SD)**<br>**B (95% CI)**
Lifetime HRT	147,119	**-0.026 (-0.036;-0.016)**	**-0.027 (-0.037;-0.017)**	87,098	**-0.045 (-0.056;-0.033)**	**-0.062 (-0.073;-0.051)**	9,819	**-0.101 (-0.152;-0.051)**	**-0.154 (-0.205;-0.103)**	3,891	-0.008 (-0.076;0.061)	-0.057 (-0.126;0.012)
Initiation HRT close to menopause [a]	35,836	-0.033 (-0.104;0.038)	-0.014 (-0.085;0.057)	31,866	-0.011 (-0.099;0.076)	-0.067 (-0.154;0.020)	1,921	0.128 (-0.363;0.619)	0.161 (-0.331;0.653)	2,049	-0.041 (-0.196;0.114)	0.139 (-0.019;0.297)

B indicates unstandardized regression coefficient; CI, confidence interval; SD, standard deviation; HbA1c, hemoglobin A1c, HRT, hormone replacement therapy. Analyses are adjusted for age and educational level. Statistically significant associations using a two-sided *p*-value < 0.05 are presented in bold. [a] Initiation <61 years or initiation <11 years from menopause for women with menopause at age >44 years or initiation <52 years for women with menopause at age <45 years.

eTable 8. Additional analyses for the associations of menopausal factors with markers of glycemia

Menopausal factors	Number of participants in analysis	Fasting plasma glucose (per 1 SD)<br>B (95% CI)	HbA1c (per 1 SD)<br>B (95% CI)
**Model 1**			
Bilateral oophorectomy aged < 50 years[a]	47,518	**0.052 (0.003;0.102)**	**0.217 (0.166;0.267)**
Bilateral oophorectomy aged ≥ 50 years[b]	95,711	**0.064 (0.044;0.083)**	**0.061 (0.042;0.080)**
Hysterectomy aged < 50 years[a]	46,561	-0.002 (-0.091;0.087)	**0.140 (0.048;0.231)**
Hysterectomy aged ≥ 50 years[b]	90,739	**0.065 (0.037;0.093)**	**0.087 (0.059;0.114)**
Age natural menopause (years)	82,426	**-0.001 (-0.003;-0.000)**	**-0.003 (-0.005;-0.002)**
Early natural menopause	82,426	**0.028 (0.008;0.048)**	**0.064 (0.045;0.083)**
Age bilateral oophorectomy (years)	9,667	**-0.008 (-0.011;-0.005)**	**-0.006 (-0.009;-0.003)**
Early bilateral oophorectomy	9,667	**0.087 (0.046;0.127)**	**0.084 (0.043;0.124)**
Age hysterectomy (age)	3,837	**-0.009 (-0.014;-0.003)**	**-0.009 (-0.014;-0.004)**
Early hysterectomy	3,837	**0.076 (0.007;0.146)**	**0.079 (0.009;0.150)**
**Model 2: model 1 + cardiometabolic factors**			
Bilateral oophorectomy aged < 50 years[a]	44,227	-0.017 (-0.067;0.033)	**0.100 (0.051;0.150)**
Bilateral oophorectomy aged ≥ 50 years[b]	89,462	0.009 (-0.010;0.028)	-0.015 (-0.033;0.003)
Hysterectomy aged < 50 years[a]	43,353	-0.044 (-0.133;0.044)	0.083 (-0.005;0.170)
Hysterectomy aged ≥ 50 years[b]	84,775	0.020 (-0.008;0.048)	0.020 (-0.007;0.046)
Age natural menopause (years)	76,946	-0.000 (-0.001;0.001)	**-0.001 (-0.003;-0.000)**
Early natural menopause	76,946	-0.003 (-0.022;0.017)	0.017 (-0.001;0.036)
Age bilateral oophorectomy (years)	9,080	**-0.005 (-0.007;-0.002)**	-0.002 (-0.005;0.001)
Early bilateral oophorectomy	9,080	**0.042 (0.002;0.081)**	0.018 (-0.020;0.056)
Age hysterectomy (age)	3,610	**-0.005 (-0.011;-0.000)**	-0.004 (-0.009;0.001)
Early hysterectomy	3,610	0.033 (-0.036;0.103)	0.016 (-0.052;0.084)
**Model 3: model 2 + health behaviour factors**			
Bilateral oophorectomy aged < 50 years[a]	33,487	-0.013 (-0.072;0.046)	**0.066 (0.008;0.123)**
Bilateral oophorectomy aged ≥ 50 years[b]	67,167	0.014 (-0.008;0.035)	-0.020 (-0.040;0.000)
Hysterectomy aged < 50 years[a]	32,891	-0.058 (-0.159;0.044)	0.062 (-0.037;0.161)
Hysterectomy aged ≥ 50 years[b]	63,826	0.028 (-0.003;0.059)	**0.031 (0.001;0.060)**
Age natural menopause (years)	58,432	0.000 (-0.001;0.001)	-0.000 (-0.002;0.001)
Early natural menopause	58,432	-0.003 (-0.025;0.019)	0.010 (-0.011;0.031)
Age bilateral oophorectomy (years)	6,574	**-0.005 (-0.008;-0.001)**	-0.001 (-0.004;0.002)
Early bilateral oophorectomy	6,574	0.045 (-0.003;0.092)	-0.005 (-0.049;0.038)
Age hysterectomy (age)	2,684	-0.004 (-0.010;0.002)	-0.003 (-0.009;0.003)
Early hysterectomy	2,684	0.017 (-0.063;0.097)	-0.007 (-0.085;0.072)
**Model 4: model 3 + deprivation**			
Bilateral oophorectomy aged < 50 years[a]	33,443	-0.013 (-0.073;0.046)	**0.066 (0.008;0.123)**
Bilateral oophorectomy aged ≥ 50 years[b]	67,101	0.014 (-0.007;0.036)	-0.020 (-0.040;0.001)
Hysterectomy aged < 50 years[a]	32,846	-0.060 (-0.162;0.042)	0.059 (-0.040;0.158)
Hysterectomy aged ≥ 50 years[b]	63,765	0.029 (-0.002;0.060)	**0.032 (0.002;0.061)**

Age natural menopause (years)	58,372	0.000 (-0.001;0.002)	-0.000 (-0.002;0.001)
Early natural menopause	58,372	-0.004 (-0.026;0.018)	0.010 (-0.011;0.031)
Age bilateral oophorectomy (years)	6,569	**-0.005 (-0.008;-0.001)**	-0.001 (-0.004;0.003)
Early bilateral oophorectomy	6,569	0.045 (-0.002;0.092)	-0.006 (-0.050;0.038)
Age hysterectomy (age)	2,683	-0.004 (-0.010;0.002)	-0.003 (-0.009;0.003)
Early hysterectomy	2,683	0.016 (-0.065;0.096)	-0.008 (-0.087;0.070)

B indicates unstandardized regression coefficient; CI, confidence interval; SD, standard deviation. Statistically significant associations using a two-sided *p*-value < 0.05 are presented in bold. [a] Compared to pre-menopausal women without surgical interference. [b] Compared to post-menopausal women without surgical interference prior to menopause. Model 1 is adjusted for age, educational level, and history of hormone replacement therapy; model 2 is additionally adjusted for waist circumference, triglyceride-to-high-density lipoprotein ratio, systolic blood pressure, use of cholesterol lowering medication, and blood pressure lowering medication; model 3 is additionally adjusted for alcohol use frequency, current tobacco use, moderate physical activity, and history of mental health problems; model 4 is additionally adjusted for deprivation.

eTable 9. Additional analyses for the association of lifetime hormone replacement therapy with markers of glycemia

	**Total study population**			**Natural menopause**			**Bilateral oophorectomy (with or without a hysterectomy)**			**Hysterectomy (without bilateral oophorectomy)**		
	**n=**	**Fasting plasma glucose (per 1 SD)**<br>**B (95% CI)**	**HbA1c (per 1 SD)**<br>**B (95% CI)**	**n=**	**Fasting plasma glucose (per 1 SD)**<br>**B (95% CI)**	**HbA1c (per 1 SD)**<br>**B (95% CI)**	**n=**	**Fasting plasma glucose (per 1 SD)**<br>**B (95% CI)**	**HbA1c (per 1 SD)**<br>**B (95% CI)**	**n=**	**Fasting plasma glucose (per 1 SD)**<br>**B (95% CI)**	**HbA1c (per 1 SD)**<br>**B (95% CI)**
**Model 1: lifetime HRT, age, educational level**	147,119	**-0.026 (-0.036;-0.016)**	**-0.027 (-0.037;-0.017)**	87,098	**-0.045 (-0.056;-0.033)**	**-0.062 (-0.073;-0.051)**	9,819	**-0.101 (-0.152;-0.051)**	**-0.154 (-0.205;-0.103)**	3,891	-0.008 (-0.076;0.061)	-0.057 (-0.126;0.012)
**Model 2: model 1 + cardiometabolic factors**	137,353	**-0.036 (-0.045;-0.026)**	**-0.047 (-0.056;-0.037)**	81,348	**-0.040 (-0.052;-0.029)**	**-0.064 (-0.075;-0.053)**	9,225	**-0.086 (-0.136;-0.036)**	**-0.124 (-0.172;-0.076)**	3,664	-0.033 (-0.102;0.036)	**-0.084 (-0.151;-0.017)**
**Model 3: model 2 + health behaviour factors**	103,370	**-0.029 (-0.040;-0.018)**	**-0.037 (-0.047;-0.026)**	61,285	**-0.033 (-0.045;-0.020)**	**-0.562 (-0.064;-0.040)**	6,653	**-0.084 (-0.143;-0.025)**	**-0.085 (-0.140;-0.029)**	2,716	-0.016 (-0.096;0.065)	-0.054 (-0.133;0.024)
**Model 4: model 3 + deprivation**	103,259	**-0.028 (-0.039;-0.017)**	**-0.036 (-0.047;-0.026)**	61,224	**-0.032 (-0.045;-0.020)**	**-0.052 (-0.064;-0.040)**	6,648	**-0.084 (-0.143;-0.024)**	**-0.083 (-0.138;-0.028)**	2,715	-0.014 (-0.095;0.066)	-0.052 (-0.130;0.027)

B indicates unstandardized regression coefficient; CI, confidence interval; SD, standard deviation; HRT, hormone replacement therapy. Model 1 is adjusted for age and educational level; model 2 is additionally adjusted for waist circumference, triglyceride-to-high-density lipoprotein ratio, systolic blood pressure, use of cholesterol lowering medication, and blood pressure lowering medication; model 3 is additionally adjusted for alcohol use frequency, current tobacco use, moderate physical activity, and history of mental health problems; model 4 is additionally adjusted for deprivation. Statistically significant associations using a two-sided p-value < 0.05 are presented in bold.

eTable 10. Additional analyses for the decomposed associations of age at natural menopause with incident dementia via HbA1c

	Number of participants in analysis (number of dementia cases)	Incident dementia B (95% CI)	Proportion mediated
**Standardized age at natural menopause (SD 1 vs SD -1)** [a]			
**Model 1**	82,426 (1,035)		
Direct		**-0.00371 (-0.00523;-0.00227)**	-
Indirect via HbA1c		**-0.00006 (-0.00010;-0.00003)**	**0.017 (0.005;0.028)**
Total		**-0.00378 (-0.00527;-0.00227)**	-
**Model 2: model 1 + cardiometabolic factors**	76,946 (950)		
Direct		**-0.00333 (-0.00487;-0.00179)**	-
Indirect via HbA1c		-0.00002 (-0.00003;0.00000)	0.005 (-0.001;0.011)
Total		**-0.00335 (-0.00489;-0.00181)**	-
**Early natural menopause (< 45 years)**			
**Model 1**	82,426 (1,035)		
Direct		**0.00357 (0.00056;0.00658)**	-
Indirect via HbA1c		**0.00018 (0.00009;0.00026)**	**0.047 (0.009;0.085)**
Total		**0.00375 (0.00071;0.00678)**	-
**Model 2: model 1 + cardiometabolic factors**	76,946 (950)		
Direct		0.00257 (-0.00044;0.00558)	-
Indirect via HbA1c		0.00003 (-0.00001;0.00007)	0.011 (-0.007;0.030)
Total		0.00260 (-0.00041;0.00562)	-

CI indicates confidence interval, SD, standard deviation. Regression results are decomposed in direct, indirect and total effects and presented as B with 95% CI. Statistically significant associations using a two-sided *p*-value < 0.05 are presented in bold. [a] Age is standardized and a standard deviation of 1 is compared to a standard deviation of -1. Model 1 is adjusted for age, educational level, and history of hormone replacement therapy; model 2 is additionally adjusted for waist circumference, triglyceride-to-high-density lipoprotein ratio, systolic blood pressure, use of cholesterol lowering medication, and blood pressure lowering medication.
